# Correction: Deciphering the chemical bonding of the trivalent oxygen atom in oxygen doped graphene

**DOI:** 10.1039/d4sc90112f

**Published:** 2024-06-11

**Authors:** Andoni Ugartemendia, Irene Casademont-Reig, Lili Zhao, Zuxian Zhang, Gernot Frenking, Jesus M. Ugalde, Aran Garcia-Lekue, Elisa Jimenez-Izal

**Affiliations:** a Polimero Eta Material Aurreratuak: Fisika, Kimika Eta Teknologia Saila, Kimika Fakultatea, Euskal Herriko Unibertsitatea (UPV/EHU) M. de Lardizabal Pasealekua 3 Donostia Euskadi Spain elisa.jimenez@ehu.es; b Donostia International Physics Center (DIPC) Manuel de Lardizabal Pasealekua 3 Donostia Euskadi Spain wmbgalea@ehu.eus; c Department of General Chemistry (ALGC), Vrije Universiteit Brussel (VUB) Pleinlaan 2 1050 Brussels Belgium; d Institute of Advanced Synthesis, School of Chemistry and Molecular Engineering, Jiangsu National Synergetic Innovation Center for Advanced Materials, Nanjing Tech University Nanjing 211816 China; e Fachbereich Chemie, Philipps-Universität Marburg, Hans-Meerwein-Strasse D-35043 Marburg Germany; f IKERBASQUE, Basque Foundation for Science Euskadi Bilbao Spain

## Abstract

Correction for ‘Deciphering the chemical bonding of the trivalent oxygen atom in oxygen doped graphene’ by Andoni Ugartemendia *et al.*, *Chem. Sci.*, 2024, **15**, 6151–6159, https://doi.org/10.1039/D4SC00142G.

The authors regret an error in the affiliation of one of the authors, Aran Garcia-Lekue, in the original article. The correct affiliations are as shown in this Correction article.

The Royal Society of Chemistry apologises for these errors and any consequent inconvenience to authors and readers.

